# An Improved Mode of Applying Caustic to Strictures in the Urethra

**Published:** 1801-06

**Authors:** Richard Cartwright

**Affiliations:** Member of the Royal College of Surgeons in London, and Surgeon to the Naval Asylum.


### *An improved Mode of applying Caustic to Strictures in the Urethra.* By RICHARD CARTWRIGHT, Member of the Royal College of Surgeons in London, and Surgeon to the Naval Asylum.

MUCH has been said on the subject of strictures in the Urethra, and great difference of opinion has prevailed with regard to the best mode of removing them; but professional men have not differed so much on the nature of the remedy, as on the practicability of limiting its application. Few, I believe, would object to the use of caustic, provided it could be conveyed to the diseased part without affecting the sound part of the canal. How far that can be accomplished by the following mode of applying it, the reader himself will be able to judge. I have only to remark, that I have been in the habit of employing it in pretty extensive private practice for several years without the misfortune of experiencing one single instance of its failure, although many of the cases had been previously, but unsuccessfully, treated in the manner recommended by Mr. Home; and that I have never witnessed a single occurrence of any of the very alarming symptoms, which have occasionally followed the application of caustic by the armed bougie. This event was naturally to be expected, if we consider the difference in the extent of surface affected by the caustic applied in the mode here recommended, and in that of Mr. Home. I need not describe Mr. Home's mode of applying caustic by the armed bougie, which is now pretty generally known. The idea of a flexible canula will immediately suggest to every one the mode I have adopted; the great difficulty has been to procure a canula made in such a way as fully to answer the purpose. The late Mr. Wyatt, so long as twenty years ago, had canulæ made of the elastic gum with the same express view, but their sides were too thick, and the calibre so small, that he could not convey the caustic up the passage in the manner he intended.

The best canula consists of a web made of the finest silk or catgut, which is afterwards covered with the elastic gum, the inside of which has also a covering of the same gum. The sides of the canula ought to be as thin as possible, possessing sufficient elasticity to counteract the contraction of the urethra, and the end of it should have a few additional coverings of the gum to prevent its scraping in its passage along the canal. This with a stilet fitted to it, (for which the common plaster bougie serves the best) and another bougie of such a size as to paſs freely through the canula, with a little lunar cauſt in fine powder, form the whole of the apparatus.

In order to avoid miſtake, it will be adviſeable to have a white bougie for the ſtilet, and a black one for the purpoſe of carrying the cauſtic through the canula, upon which the quantity of cauſtic will be more readily diſcerned.

The ſtilet ſhould fill up exactly that end of the canula which is to be introduced; and this it can eaſily be made to do, by moulding it a little with the fingers; the black bougie ſhould be cut off at the broader end, that it may have the greateſt flat ſurface, and on the other end a mark ſhould be made to correſpond with the length of the canula.

The manner of applying the cauſtic is then very ſimple. Paſs the canula, fitted with its ſtilet, gently down to the ſtricture; withdraw the ſtilet, taking care to keep the canula ſteady in its place; then dip the flat end of the black bougie into the powdered cauſtic, ſo that the ſurface may be covered, and apply it with as much celerity as poſſible to the ſtricture, which application may be repeated two or three times, or more, as the operator may think proper.

In cold weather the black bougie will require to be warmed a little, in order to make the cauſtic adhere to its ſurface for the firſt application.*

The ſeparation of the ſlough depending on the action of the part to which the cauſtic has been applied, will take place at different times under different circumſtances. It will, however, ſeldom happen in leſs than two days, about which time I generally try to paſs a bougie; and, ſhould there be pus formed, I go on with the daily uſe of bougies alone, as long as the ſtricture dilates and the ſuppuration continues; but, ſhould the ſuppuration ſtop before the ſtricture is removed, it will then be neceſſary to have recourſe again to the uſe of the cauſtic.

The advantages which this mode of applying cauſtic to ſtrictures in the urethra has over that by the armed bougie, are obvious. By means of the canula the cauſtic may be conveyed with great facility to any part of the canal, and may be as confined in its application as the operator may wiſh, however great the irritability of the urethra may be; and as to the pain

* The canula after being uſed, ſhould be waſhed with cold water, wiped, and left to dry on a braſs wire for a day or two, and ſhould then be beſmeared inſide and outſide with a little oil; and with theſe precautions one canula will ſerve for ſeveral hundred applications. Canulæ of all ſizes may be had of Mr. Savigny, Surgeons' Inſtrument Maker, King Street, Covent Garden, London; and of Mr. Carlberg, Great Windmill Street, London.

pain occaſioned, the patient rarely feels more than is experienced in paſſing a common bougie, and frequently leſs. The cauſtic may thus be applied, of its full ſtrength, undiluted by mucus, blood, or urine. The ſlough from ſo partial and limited an application is neceſſarily ſmall, the inflammation very inconſiderable, and the hæmorrhage, if any, ſeldom exceeds a few drops of blood. I have never met with a caſe of ſwelled teſticle from the application of cauſtic through the canula. In ſome few inſtances there has been a ſlight ſuppreſſion of urine, which has always ſoon ſubſided under the cuſtomary remedies. This has been the greateſt inconvenience that enſued even in patients who indulged in every irregularity and exceſs of free living.

I am well convinced, from ample experience, that a patient labouring under ſtricture runs more riſk in ſubmitting to the treatment by bougies alone, than by cauſtic introduced through the canula. This may be accounted for from the inflammation attending the uſe of bougies extending further than that from the application of cauſtic, of which there can be little doubt in old obſtinate ſtrictures.

The public would long ago have been in poſſeſſion of this mode of applying cauſtic, had not many difficulties interpoſed in bringing the canula to its preſent ſtate of perfection.

It was my intention to have given a ſelection of the moſt intereſting caſes of ſtrictures which have fallen under my care, with practical obſervations on ſome effects of Strictures not hitherto noticed, as well as on the comparative utility of the common plaſter bougie and that of the elaſtic gum, which is now made of ſuch a pliability as readily to accommodate itſelf to the curvature of the urethra, and of ſufficient hardneſs to reſiſt the action from ſtricture. This I ſhall, however, reſerve to form the ſubject of a ſeparate publication at ſome future day.

### POSTSCRIPT.

Since writing the above, a publication by Mr. Whately has fallen into my hands, propoſing an Improvement of Mr. Home's Plan of applying Cauſtic. The following are his directions how to prepare the bougie for that purpoſe. "Touch an eighth, or from that to a quarter of an inch, of the end of a bougie of any ſize, with a ſmall bruſh dipped in common glue, as uſed by mechanics; and let the coating of glue be as thin as poſſible. The glued end muſt be immediately applied to a given quantity of powdered lunar cauſtic, put upon a piece of writing paper; this ſhould be done by alternately putting its different ſides to the cauſtic, until the whole of it

 adheres.

adheres. The bougie in this ſtate muſt be laid in ſome dry place to harden, which effect will take place in a few hours. When it is ſufficiently hardened, the glued end muſt be gently rolled to and fro upon a table, with a bit of ſmooth wood, about four inches ſquare, till it is perfectly level and ſmooth. If the bougie be very hard, or the weather cold, this end muſt be previouſly warmed a little by the fire. The part thus covered with cauſtic ſhould then be very lightly rubbed with a bit of bees' wax, with the intention of giving it a very thin coating of this ſubſtance. After this, let it be kept for uſe in a glaſs veſſel well cloſed."

Now, admitting even that the cauſtic could really be applied, *of its full strength*, in this way, there is one very obvious and inſuperable objection to this plan; that is to ſay, the miſchievous conſequences which muſt neceſſarily ariſe, on withdrawing the bougie, in the ſound part of the urethra, when the cauſtic is ſet at liberty by the ſolution of the glue. But as none of theſe miſchievous conſequences are ſtated by Mr. Whately to have enſued, I ſuſpect that he has deceived himſelf in ſuppoſing that he has been applying the cauſtic *in its full strength*, which we know muſt certainly have been conſiderably weakened in its corroſive properties by its admixture with the glue, and its expoſure to the atmoſphere.

